# Greetings

**Published:** 1882-12-20

**Authors:** 


### EDITORIALS AND MISCELLANEOUS.

### *GREETINGS.*

We are pleased to acknowledge the many notes of commendation and encouragement received from our readers—their almost unanimous acceptance of our proposition for renewal the coming year, and to be able to promise, on our part, renewed zeal and energy in presenting the medical news in the future. We trust that our friends will stick to us, as brethren in a common cause, and that all in arrears will promptly settle up, giving us a good start with the new year. May they also take an interest in our Journal as an organ of Southern medical literature, contribute to our pages, and aid us in extending our circulation.

Remember our liberal proposition, to accept of our subscribers on renewal $3.00 for two copies; that is, each subscriber who, on renewing his subscription for 1883, will send us a new name, may deduct 50 cents from his own subscription and that of his friend. Help us to increase our list, and in so doing you will enable us to improve the Journal, and thus do a good work for yourselves and for the Medical Literature of your section. With these remarks we extend fraternal regards and Christmas greetings to all of our readers.

